# Pathological narcissism and inceldom: can the application of treatment principles for PN help reduce the rise of incel-related incidents?

**DOI:** 10.3389/fpsyt.2025.1513719

**Published:** 2025-05-30

**Authors:** Acelya Akdag, Robert Blakey

**Affiliations:** Department of Systems Medicine, Faculty of Medicine and Surgery, University of Rome Tor Vergata, Rome, Italy

**Keywords:** incels, andrew tate, elliot rodgers, misogyny, pathological narcissism, aggrieved entitlement, hegemonic masculinity

## Abstract

**Introduction:**

Involuntary celibates, henceforth known as incels, are individuals unable to secure sexual or romantic partners, resulting in feelings of anger towards women. Within the ‘manosphere’, incel radicals like Elliot Rodger are often idolised for perpetrating misogynistic terrorist acts. While interdisciplinary research within sociology, criminology and political science has surged, a significant gap remains in exploring underlying psychological contributors of incel-related beliefs. There are notable parallels in the cognition, attitudes andbehaviours between individuals with Pathological Narcissism (PN) and incels.

**Method:**

This systematic review explored the intersection between the two populations, to gauge whether intervention principles designed for PN might be applicable in mitigating incel-related beliefs and behaviours. A comprehensive search across multiple databases identified 12 studies, both quantitative and qualitative, that met the selection criteria.

**Results:**

A narrative synthesis revealed emerging themes of sexual frustration, perceived male oppression, aggrievedentitlement and hegemonic masculinity.

**Discussion:**

Future empirical endeavours should investigate whether the identification of PN in incels could serve as a predictivemarker for incel-related radical beliefs and behaviours.

## Introduction

At the time of writing, newly appointed British Prime Minister Keir Starmer has declared violence against women and girls a national emergency. A recent National Police Chiefs’ Council report estimates that approximately two million women and girls in the United Kingdom alone are subjected to male-perpetrated violence yearly, illustrating the severity of this problem, despite its recent recognition as urgent (1). A tragic example is Elianne Andam, a 15-year-old girl fatally stabbed by a 17-year-old boy in September of 2023, after rejecting his romantic advances (2). This raises the question: why did this romantic rejection provoke such a violent response, culminating in the loss of a young girl’s life? This prompts an urgent inquiry into potential underlying factors that may explain such an extreme reaction, in an effort towards preventing further tragedies.

It is essential to consider the current climate of misogyny. In 2022, Andrew Tate, a social media personality known for promoting a lifestyle centred around hypermasculinity, luxury, and control over women, gained widespread attention (3). His controversial, far-right views on women’s subjugation and toxic masculinity has circulated widely across mainstream media. Tate positions himself as a solution for young men who feel they have failed to achieve ‘true’ masculinity, offering online courses claiming to teach traditional masculinity, ‘stoicism’ and paths to wealth. Though, his teachings arguably promote the domination of women rather than stoicism (3). Despite multiple arrests for rape and sex trafficking, Tate remains influential, particularly among young males (4). Although deplatformed by major platforms such as TikTok, Tate frames his ‘cancellation’ as being silenced for speaking the truth, further reinforcing his followers’ support (3).

The glorification of ‘traditionally masculine’ figures by young males can profoundly distort their views on gender, particularly given the rapid expansion of media and communication. One such figure is Elliot Rodger, a self-identified incel, who articulated his hatred of women in his manifesto, “My Twisted World”, released prior to carrying out a terrorist attack, where seven people, including himself, were killed. Rodger stated, “I would take great pleasure and satisfaction in condemning every single woman on earth to starve to death” envisioning a world without women and positioning himself as “the true victim” (5, p. 136-137). He lamented “How dare those girls give their love and sex to those other men but not me?” (5, p.134). Rodger has since become an idol among self-identifying incels, with his misogynistic campaign guiding others in navigating their emotions and subsequent behaviours (6). Although not all incels commit such violence, it has inspired others to replicate his homicidal actions like Alek Minassian in his honour (7, 8).

## Inceldom

The concept of “inceldom” originated in 1997 when student Alana created “Alana’s Involuntary Celibacy Project” to share her dating struggles and establish a supportive online community (9). Inceldom in its contemporary form emerged in the late 2000s as a subculture within the broader “manosphere”. Today, inceldom comprises online communities of heterosexual men united by their shared desire for romantic and sexual relationships yet consistently fail in attaining them (10, 11). They often attribute this failure to factors such as their physical appearance, neurodivergence and lack of social popularity (12). This perceived failure often leads to scapegoating women, whom incels regard as the gatekeepers of sex and relationships, resulting in the endorsement of misogynistic abuse (10). Incels weaponise their perceived victimhood to oppose feminism, as they believe the feminisation of society through the evolution of gender equality has created societal oppression against men (10, 13, 14). Inceldom has thus transformed into an echo chamber for extremist ideologies, espousing antifeminist and white male supremacist beliefs (14), often expressed through their distinct lexicon using derogatory terms like “feminazi” (6, 15, 16).

The COVID-19 lockdown intensified this issue, as young men seeking connection amidst loneliness gravitated towards the manosphere, particularly online platforms like Reddit (12). Within these spaces, insecurities are validated, frequently escalating into the weaponisation of perceived victimhood (17). This shared sentiment cultivates a unified opposition to feminism, asserting claims of women inferiority, akin to the ‘Beauvoirian other’ (18). The incel community’s reverence for idols like Rodger, who simultaneously eroticise women while subjecting them to intense hatred, underscores the oscillating attitudes toward women. This perpetuates a cycle of hatred that is both dangerous and deeply rooted in their distorted understanding of gender relations (7). Such polarisation is also reflected in how incels perceive traditional standards of masculinity.

Literature in this field often adopts Connell’s Hegemonic Masculinity Framework to explore the incel experience (19). It postulates that men establish ‘traditional’ ideals to structure society in gender-unequal ways, by preserving male power and dominance over non-hegemonic others, such as women or non-masculine men (3, 8, 19). Incel ideology reflects this framework through an attractiveness-based hierarchy: ‘Chads’, representing white, masculine, attractive men, occupying the top; ‘normies’ the middle; and incels the bottom, claiming discrimination by ‘Stacys’, or attractive women (10, 20). Incels view themselves as victims in today’s ‘feminised’ society, perceiving a loss of their rightful power to women (3). A sense of entitlement facilitates their victimhood, as they believe their white, male identity inherently entitles them to sexual and romantic relationships (17, 21). Kimmel and Kalesh's (21) concept of “aggrieved entitlement” captures the dissonance between their perceived rightful status in society and their actual position. Denial of this entitlement may provoke retaliatory efforts to reestablish their hegemonic masculinity. This behaviour underscores the link between hegemonic masculinity and aggrieved entitlement, with masculinity being identified as a significant risk factor (21, 22). The resulting violence may serve as over-compensation for perceived deficiencies in masculinity, particularly among belittled or bullied men, like incels. Such displays of aggrieved entitlement are reminiscent of Rogers’ self-concept theory, where incongruence between one’s ideal and real self can fuel frustration, distorted self-perception, and intrapersonal conflict (8, 23, 24), thus offering insight into how this dissonance can trigger such harmful reactions.

Such manifestations of entitlement, self-criticism, and superiority reveal noteworthy parallels between inceldom and PN. This paper concentrates on PN, as opposed to Narcissistic personality disorder (NPD) to differentiate pathology from normal variation. Additionally, it offers a more comprehensive conceptual framework than NPD, encompassing multiple dimensions, including both narcissistic vulnerability and grandiosity, as well as overt and covert presentations (25).

## Pathological Narcissism

PN merged as a contemporary psychological phenomenon particularly within Western societies (26, 27). The increasing glorification of figures like Tate highlights the urgency of addressing PN, given the media’s influential role in shaping narcissistic tendencies, especially among impressionable young males (28). Despite its growing prevalence, there is a paucity of empirically supported interventions (25, 29), exhibiting the need for immediate and focused research efforts.

The conceptualisation of PN encompasses both overt, grandiose narcissism and covert, vulnerable narcissism (30), elucidating its nuanced phenomenology (31). Grandiose narcissism (GN) is marked by exhibitionism and feelings of superiority, whereas vulnerable narcissism (VN) involves hypersensitivity to criticism and diminished self-esteem (27, 32, 33). Nevertheless, both share certain underlying traits, particularly deficits in empathic functioning (34). Individuals with PN often exhibit poor reflective processes, including theory of mind, contributing to difficulties in emotional regulation and interpersonal relations (31, 35). Diagnosing both PN and NPD presents challenges as the ICD-10 does not recognise NPD as a distinct diagnosis, while the DSM-5 categorises NPD as only moderately impairing compared to other personality disorders, raising concerns about its clinical significance (30). The DSM-5 characterises NPD as a “pervasive pattern of grandiosity, a need for admiration and a lack of empathy” (36, p. 669). Individuals with NPD often measure their self-worth on achievements, leading to negative self-perception and self-criticism when they fail to meet their self-imposed standards of success (37, 38). The diagnosis requires the presence of five or more traits, including grandiosity, preoccupation with fantasies of unlimited success, beliefs of uniqueness, a need for excessive admiration, entitlement, interpersonal exploitive behaviour, envy, and haughtiness (30, 35).

Narcissism is conceptualised through various models, offering distinctive perspectives. While NPD, a single-factor model, is traditionally understood through two primary dimensions—GN and VN—the more recent Three-Factor Model of Narcissism (3FM) provides a broader framework. The single-factor model fails to explain the oscillating presentations of both dimensions (39). The 3FM, encompassing agentic extraversion, neurotic narcissism, and self-centred antagonism, has demonstrated validity in measuring PN and captures its diverse manifestations (33). Central to this model is antagonism, the core characteristic underlying both GN and VN. Antagonism involves power struggles, interpersonal difficulties, self-esteem instability, and entitlement (40). Narcissistic neuroticism is most closely associated with VN and emotional instability, while agentic extraversion is characterised by low levels of agreeableness (33, 39). Additionally, these three factors integrate key qualities associated with the Dark Triad traits—narcissism, Machiavellianism, and psychopathy— therefore offering a more parsimonious classification of narcissistic expressions (39).

Individuals exhibiting PN demonstrate impaired cognition that contributes to dysfunctional behavioural patterns (41). A prominent maladaptive cognitive style observed in this population is dichotomous thinking (42). Characterised by a black-and-white approach, it is frequently employed as a problem-solving strategy offering a deceptive sense of clarity, especially in decision-making. This thinking strongly correlates with Cluster B personality disorders, particularly NPD (43), and therefore may help yield valuable insights into the binary self-perceptions typical of narcissistic individuals. Dichotomous thinking may serve as a strategy to preserve their idealised self-image or, conversely, perpetuate negative self-perceptions of failure (43–45). Such self-images may also be linked to other distorted thinking styles, like rumination, characterised by repetitive negative thought patterns. Rumination in narcissistic individuals may significantly exacerbate narcissistic tendencies, though its connection is less established compared to other personality disorders (33, 46). Social comparative thinking is also observed in individuals with PN, particularly downward comparisons, wherein they compare themselves to those they perceive as inferior to bolster their self-esteem. While less frequent, engaging in upward comparisons tend to evoke feelings of envy and hostility toward perceived superiors (38).

Multiple factors contribute to the development of PN, with extreme parenting styles, namely parental overvaluation and cold caregiving, being a consistent theme in the literature (47). Parental overvaluation, characterised by excessive involvement and appraisal, fosters a child’s sense of uniqueness and dependence on external validation, predictive of GN (48, 49). Conversely, cold parenting, marked by emotional neglect, parental invalidation, and contradictory messages, has been associated with VN, characterised by affective instability and hypersensitivity to rejection (29). When caregivers respond incongruently to a child’s affective states, the child may internalise a distorted self-concept, what Fonagy and Target (50) term the “alien self.” This alien self functions as a defensive structure against early emotional neglect, obstructing the development of mentalisation- the capacity to understand one’s own and others’ behaviours through underlying mental states. Narcissistic traits may thus emerge as compensatory coping mechanisms to manage unmet needs for admiration, attention and emotional support (31, 51, 52).

A psychoanalytic perspective offers a nuanced understanding of how parenting patterns may contribute to aggression in PN. Kohut’s self-psychology posits that when caregivers do not function as reliable in the marked mirroring (i.e., self-referential processing) of the child’s emerging self, or self-objects, this results in a fragmented self-structure (53, 54). To defend against this, the child may construct a grandiose self, sustained through idealisation and external admiration. Kohut introduced narcissistic rage to describe the intense emotional responses triggered by perceived threats to self-cohesion, such as humiliation or parental invalidation, reflecting a desperate effort to manage the internal chaos.

In contrast, Kernberg’s object relations theory views PN as the outcome of a dysfunctional personality structure. It proposes that cold or inconsistent parenting disrupts the child’s ability to integrate positive and negative aspects of the self and others, resulting in defences such as splitting and devaluation (55). Consequently, the individual becomes fixated on a grandiose self-image, while perceiving others as either idealised extensions or threatening competitors. Kernberg labelled this as malignant narcissism, characterised by sadism, paranoia, and interpersonal exploitation. Overall, narcissistic traits may function as maladaptive strategies compensating for early relational disruptions, specifically extreme parenting styles.

There exists a notable intersection between the incel community and individuals with PN, particularly in their shared tendency to glorify certain idols. Twenge and Campbell (26) assert that “Americans are obsessed with people who are obsessed with themselves.” In contemporary Western society, although the self-esteem movement is widely regarded as empowering, it could be contributing to the rise of PN (56). The current generation’s fixation on fame has elevated arrogant public figures as role models for younger individuals. Research exhibits that exposure to reality television featuring overtly narcissistic personalities correlates positively with increased narcissism among viewers (28). These observations underscore the media’s significant influence in shaping narcissistic tendencies (28), particularly in the current online landscape, where the glorification of figures endorsing harmful ideologies is prevalent.

Although empirically supported interventions targeting PN is limited, established treatment principles are endorsed by leading clinicians. These practitioners emphasise the importance of the therapeutic alliance and a supportive therapeutic environment to help clients positively reframe distorted thinking. Techniques from metacognitive interpersonal therapy and transference-focused psychotherapy are frequently utilised to address negative thought patterns, enhance awareness of cognitive processes and promote healthier perceptions and responses (25). Individuals with PN often face significant difficulties in interpersonal relationships, coping strategies and emotion regulation (25, 31). These challenges can also contribute to clinician burnout, exacerbating the scarcity of effective treatment options. Thus, equipping clinicians with effective strategies is essential in reducing the risk of patient dropout and clinician burnout.

The growing interdisciplinary literature on inceldom reveals emerging patterns of aggrieved entitlement, self-criticism, and adherence to hegemonic masculinity, closely parallelling PN characteristics such as grandiose fantasies, entitlement, and hypersensitivity to criticism (31, 57). Although most incels do not endorse violent attitudes (10), the alarming increase in violence against women and girls necessitates urgent attention to the incel community to address the rise in incel-related incidents. Given these parallels, this systematic review adopts a psychological perspective, offering nuanced insights into the incel experience and exploring strategies to address incel-related violence. This approach is particularly critical given the current paucity of psychological research on incels which could highlight predictors of incel-related behaviours for prevention (58).

The aims of the current paper:

1. To highlight the overlap between the characteristics and behaviours of individuals with PN and self-identified incels
2. To explore whether effective interventions and tools used for PN may be applicable to incels to prevent the development of harmful, radical beliefs into potential incel-related attacks.

## Method

This systematic review aims to explore the potential overlap between PN and inceldom, and how treatment principles for PN may be potentially applicable to incels. A systematic review has been used to collate the existing research, in an operationalised and replicable manner, guided by the PICO (Population, Intervention, Comparison, Outcome) framework. A pilot study was conducted which ensued revisions, initially focusing on qualitative research for its idiographic data regarding the lived incel experience. Following the pilot, both qualitative and quantitative methods have been included to widen the search and address the challenges in obtaining sufficient research.

### Search strategy

The studies that were selected for the systematic review were searched and found on the following electronic databases:

- Scopus – selected for its interdisciplinary coverage of peer-reviewed literature across the social sciences and humanities, ensuring a comprehensive search
- The Cochrane Library – selected for research on narcissistic psychopathology and mental health interventions, relevant to understanding incel-related behaviours
- APA PsychArticles – selected for its peer-reviewed psychology journals, providing sources on both PN and the psychosocial underpinnings of inceldom

### Search terms

The following search terms (see **Table 1**) reflect the selection criteria and were entered into the aforementioned databases:

**Table 1:** Search terms.

Search terms
Narcissistic personality disorder OR Pathological Narcissism AND Grandiosity OR lack of empathy OR Fantasies OR Entitlement OR Loneliness OR Thinking Styles AND therapeutic interventions OR therapeutic strategies OR therapeutic techniques. OR Incel OR Inceldom OR BlackPill OR Elliot Rodger OR Andrew Tate AND Violence OR Misogyny OR Homicide OR Suicide OR Loneliness OR Idolisation.

**Selection criteria:**

The selection criteria followed the PICO (population, intervention, comparators, outcome) framework to ensure relevancy to the aims of this review (see **Table 2**). Studies were selected if the population consisted of participants over the age of 18, who identify as incels, either by self-identification or validated scales, namely the incel traits scale (59). Studies included individuals diagnosed with PN or NPD according to DSM-5 criteria (36), or those presenting symptomatic narcissism as part of other conditions, like the dark triad, to widen the search and to compare the two populations. Studies with outcomes focusing on non-pathological narcissism were excluded. Participants were identified through narcissism measures, such as the Narcissistic Personality Inventory (60–63). Participants with comorbidities and co-morbid symptoms of NPD, such as depression were included to account for the prevalence of comorbidities and to increase the findings’ generalisability (64). Studies were excluded if they involved participants under the age of 18, as the focus was on adult romantic/sexual relationships. Female incels, or Femcels, were also excluded as their experiences differ and fall outside the scope of this research (65).

**Table 2:** Summary of population, intervention, comparison, outcome and study design components.

PICOS elements	Included	Excluded
P	PN or NPD Comorbid conditions or symptoms including anxiety or depression Ages 18+	Individuals under age 18 Non-pathological narcissism
I	Treatment suggestions for PN/NPD from multiples interventions such as CBT, DBT, MIT and MBT	Treatment that is not suggested for PN or NPD
C	Self-identified incels	Femcels
O	Studies investigating incel phenomenology Studies investigating the presentation of PN or NPD Studies investigating therapeutic progress: Improvement in symptomatic presentation and daily functioning after treatment for PN or NPD	Research published before 10 years ago Papers not reporting outcomes related to presentations of PN or incel experience
S	Grey literature Quantitative research, including cross-sectional studies and Qualitative research, including interview designs	

Studies examining the therapeutic progress of narcissistic individuals, such as therapeutic techniques from transference-focused psychotherapy (TFP), mentalisation-based therapy (MBT), cognitive-behavioural therapy (CBT), schema therapy (ST) or psychoanalytically oriented therapy were included as these are commonly recommended for PN/NPD (66). Studies also included individuals without PN and non-incels as comparator groups to evaluate differences both between these populations and the general population.

The inclusion criteria also extended to non-English studies, provided they were translated, to broaden the search. Grey literature, quantitative and qualitative studies were included to provide a holistic view by exploring the nuances of incels’ and narcissists’ experiences, whilst measuring attitudes using standardised assessments. Studies published over ten years ago were excluded to reflect the evolving incel landscape, which has shifted significantly from Alana’s original purpose (13).

These criteria ensure that the systematic review focuses on the most relevant evidence. By including diverse study designs, the review can capture an array of insights regarding both narcissism and inceldom, whilst maintaining the review’s focus.

**Data extraction:**

The systematic search involved entering the search terms into the databases to gather research. The results of each search were then screened by deleting duplicate papers and papers with irrelevant titles, via the use of the artificially intelligent software Rayyan. At the second stage, the abstracts were read to ensure compatibility with the selection criteria. The remaining papers were then read in full to assess alignment with the PICO framework.

### Quality assessment

This paper conducted a systematic review on research with varied methodologies, including journal articles, case reports and grey literature, both qualitative and quantitative, due to the limited research in this field. Four assessment checklists were used to screen the quality of research.

The Critical Appraisal Skills Programme (67) assessed the quality of the validity and the significance of the selected qualitative studies (check **Appendix 1**), including an interview design and qualitative analyses. This tool assesses elements such as the research focus, the relevancy of the data collection and the rigour of the data analysis.

The Joanna Briggs Institute (JBI) Critical Appraisal Checklist assessed the quality of case reports (check **Appendix 2**). This tool evaluated the sources by assessing the clarity of the outline of the patients’ diagnoses, presentations and interventions (68).

The JBI Critical Appraisal Checklist for Analytical Cross-Sectional Studies (69) assessed the quality by ensuring sufficient clarity and detail to ensure replicability and synthesis of the reported findings (check **Appendix 3**). The tool emphasises the significance of the study sample, reliable measures and statistical analysis. The adherence to the JBI checklist ensures comprehensive reporting of the findings.

Additionally, the JBI Checklist for Textual Evidence: Expert Opinion (70) was used to critically appraise the quality of expert opinions (check **Appendix 4**) by evaluating the clarity of the opinion, the expert’s standing in their field and its congruence with the extant literature.

### Data synthesis

Once the sources were screened and extracted, descriptive characteristics of the sources were presented in a table, describing the population, intervention, comparators, outcome/s and the study designs. The sources were then detailed using a narrative synthesis to outline the key findings by grouping the overarching themes. Finally, a discussion exhibited the relations of the sources with each other and with the aims of the review at hand.

### Ethical considerations

Ethical considerations were central role to this project, given the topic’s sensitivity. Considering the stigmatising potential of terms such as “incel”, particular care was taken to use descriptive rather than judgemental language, to avoid contributing to misunderstanding or marginalisation. Efforts were made to present findings in a balanced and evidence-based manner, recognising the variation and complexity within the incel community and among individuals exhibiting PN.

## Results

**Figure 1:**
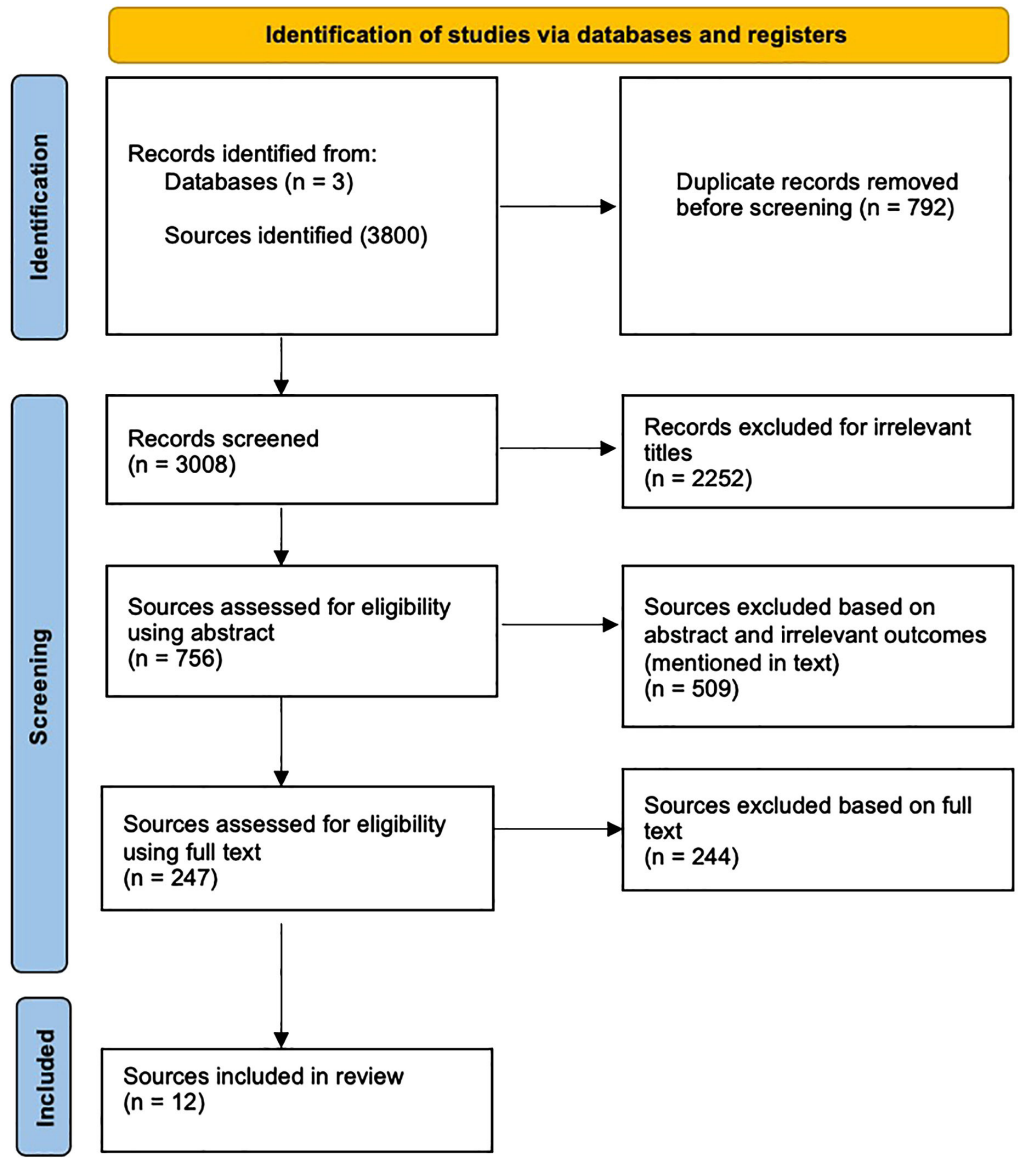
Systematic search according to (PRISMA) guidelines (73).

The searches of the databases collectively retrieved 3800 results. Initially, the duplicate papers and irrelevant titles were removed, like those not specifically addressing incels or PN/NPD. The remaining abstracts were then reviewed to assess the relevancy. 509 irrelevant papers were excluded, including focuses on sub-factions like “NoFap,” which fell outside this review’s scope (e.g. 71). Quantitative and qualitative sources were both included to provide significant insights into incels, PN and potential therapeutic strategies. Additionally, numerous studies were excluded for irrelevant outcomes, for example, NPD and substance dependence (e.g. 72). A detailed PRISMA (Preferred Reporting Items for Systematic Review and Meta-Analyses) flow diagram (73), outlining the stages and reasons for exclusion, is provided in **Figure 1**. After reading the remaining texts in full, 12 studies were selected for this review for their relevancy.

### Study characteristics

### Participant characteristics

The selection criteria specifies that the population must be over the age of 18 as attitudes regarding romantic and sexual relationships are being analysed. The highest range of age for the participants was 18–72 years (1), however, not all studies provided the average age. All studies selected have both male and female participants, however only male incels were included. In total, 38.44% of participants were male (*N* = 911). Additionally, 105 participants overall were incels (9, 10, 11).

The quantitative studies often recruited participants from student populations, offering incentives like course credits (2, 3, 4, 5, 11). One study recruited participants via Reddit, based on their incel-related posts (9). The sample sizes varied significantly, ranging from 1 to 532 participants. One study conducted an analysis of a subreddit with over 30,000 subscribers (10). Three studies specifically included incel participants (9, 10, 11). Daly and Reed (10) had a relatively small sample (*N* = 10), but the authors justified this by highlighting the hidden nature of the incel population and the appropriateness of a qualitative methodology. Similarly, Konutgan (58) included a small incel sample (*N* = 28) and noted recruitment challenges due to a five-week data collection period, thus also including non-incels to supplement the sample.

Participant characteristics varied, particularly regarding diagnoses of NPD or PN, with frequent comorbidities such as mood disorders (4, 6). Only one study inquired whether participants were currently seeking mental health treatment (4), though two additional sources focused on case studies of patients receiving treatment (6, 8) (see **Table 3**).

**Table 3:** Study characteristics of studies regarding pathological narcissism.

Study	Population	Intervention	Comparison	Outcome
1. Bonfá-Araujo and Hauck Filho (74)	*N* = 448 M age = 29.54 24.4% male	N/A	N/A	Dichotomous thinking (DTI score) positively correlated with narcissism (NPI score) but not significant (*r* = .10).
2. Campbell et al. (75)	Experiment 1: *N* = 160 50% male Undergraduates Experiment 2: *N* = 64 24.38% male undergraduates	N/A	Non-narcissists	1. NPI scores (*M* = 16.27, *SD =* 7.15). A significant interaction between feedback type, narcissism and dependent measures of relationship closeness and attribution of responsibility, *F*(1, 152) = 8.42, *p* <.004. Narcissists* took more credit for success (PV = .58) compared to failure (PV = −.46), (*b* = .32, *t*(155) = 2.85, *p* <.005). Non-narcissists took less credit for success (PV = −.39) and more responsibility for failure (PV = .37), (*b* = −.23, *t*(155) =−2.09, *p* <.039, *b* = −.23). Narcissists assigned more importance to success (PV = 0.49) than failure (PV = −.55), with this difference being statistically significant [*b* = .22, *t*(155) = 2.06, *p* = .041]. Non-narcissists also assigned more importance to success (PV = .85) than failure (PV = −.91), (*b* =.39, *t*(155) = 3.48, *p* <.001). 2. NPI scores (*M* = 17.08, *SD* = 7.03) Narcissists took significantly more responsibility for success (PV = .69) than failure (PV = -1.26), [*b* = .57, *t*(59) = 3.50, *p* <.001)]. Non-narcissists also took more responsibility for success (PV = .82) than failure (PV = -.31), [*b* = .35, *t*(59) = 2.12, *p* <.038]. Narcissists attributed more importance to success (PV = 1.26) than to failure (PV = .46), but not significant [*b* = .31, *t*(1, 60) 5 2.57, *p* <.013]. Non-narcissists also attributed more importance to success (PV = .53) than to failure (PV = -1.36), *t*(59) = 1.88, *p* <.065).
3. Ksinan and Vazsonyi (32)	*N* = 532 M age = 23.33 45.1% male	N/A	N/A	SIAS** and SSE were highly correlated (*r* = −.69). POSI was significantly, negatively associated with SSE (*r* = −.39) and positively associated with SIAS (*r* = .49). Significant positive relationship found between POSI and VN (β = .44) and a negative relationship with GN (β = -.12). POSI also significantly negatively associated with SSE (*r* = -.39). Both GN (β = .37) and VN (β = .35) associated with SIAS. Significant indirect effect of VN through POSI on SIAS (β = .094, *p* <.001). But not GN on SIAS (β = −.022, *p* <.06). VN had a significant indirect effect through POSI on SSE (β = −.087, *p* <.001), but GN was not significant (β = .021, *p* <.06). No association found between narcissism subtypes.
4. Mason and DeShong (33)	*N* = 362 M age = 19.65 Students 32.3% males 15.2% taking meds for psychological disorder 16% currently seeking mental health treatment	N/A	Comparing the relationship of the three-factor model of narcissism (agentic extraversion, narcissistic neuroticism and self-centred antagonism) and single factor model (NPD) with four types of repetitive negative thinking (general rumination, anger rumination, worry, catastrophising)	Agentic extraversion negatively correlated with worry, with a small effect size (-.149, *p* <.01). Narcissistic neuroticism had a large positive correlation with worry (.572, *p* <.001), a moderate positive correlation with catastrophising (.127, *p* <.01) and general rumination (.348, *p* <.001), and a small positive correlation with anger rumination (.272, *p* <.001). Self-centred antagonism had a moderate positive correlation with anger rumination (.367, *p* <.001), a small positive correlation with catastrophising (.127, *p* <.01) and a small negative correlation with worry (-.180, *p* <.01). The NPI-21 total score had a small significant negative correlation with worry (-0.240, *p* <.001).
5. Zeigler-Hill et al. (76)	*N* = 442 M age = 20.92 21.27% males	N/A	N/A	Different narcissism subscales were associated with different maladaptive early schemas GN and VN associated with entitlement (β = .12, *p* < 0.05, β = .13, *p* <.05 respectively) NPI Superiority associated with negatively associated with isolation (β = −.35, *p* <.001) NPI Self-admiration associated with unrelenting standards (β = −.21, *p* <.001)
6. Centonze et al. (77)	*N* = 1 Laura 38-year-old female diagnosed with covert narcissism, generalised anxiety disorder, depression and complicated grief	Metacognitive interpersonal therapy	N/A	Symptomatic improvement and improvement in narcissistic behavioural patterns after two years Improvements included better self-esteem, self-reflection, healthier self-image and interpersonal functioning
7. Dimaggio (25)	Previous clients with PN and NPD	Integrated therapy using concepts from therapies including CBT, ST, MIT, MBT and TFT	N/A	Treatment suggestions like the therapeutic alliance and contract, experiential techniques like behavioural assignments due to lack of empirically supported interventions
8. Weinberg (27)	N = 1 Matthew, early twenties Individuals with NPD and PN	Principles from psychoanalytically oriented psychotherapy, TFP, MBT, MIT and formulation of general principles for NPD	N/A	Mechanisms of change in psychotherapies: therapeutic alliance, reflective function, mourning Modification of effective treatments for related conditions Eclectic treatments Development of treatments that target mechanisms of change

N/A abbreviation for Not applicable.

*Narcissists are calculated as 1 SD above the mean, non-narcissists are 1 SD below the mean.

**SIAS abbreviated for Social interaction anxiety scale, SSE abbreviated for social self-efficacy.

### Intervention characteristics

Three qualitative reports were focused on intervention suggestions for PN and NPD, where comorbid conditions like anxiety disorders were also present (6, 7, 8).

Centonze et al. (77) evaluated the effectiveness of MIT, particularly the use of experiential techniques, and reported significant progress after two years of treatment.

Dimaggio (25) outlined treatment principles for PN and NPD from various approaches including MIT, CBT, TFP, MBT, ST and DBT.

Weinberg (27) similarly focused offered recommendations for PN and NPD, derived from TFP, MBT, MIT, POT and general principles for the treatment of NPD and PN, using a case study of 20-year-old Matthew (see **Table 4**).

**Table 4:** Study characteristics for studies regarding inceldom.

Study ID	Population	Intervention	Comparators	Outcome
9. Daly and Reed (10)	*N* = 10 incels *M* Age = 23.6	N/A	N/A	Themes found were masculinity challenges, subhuman status and societal rejection, the BlackPill, shitposting and perceived effects of inceldom
10. Glace et al. (15)	Content analysis of Incel posts from R/Braincels	N/A	N/A	Themes extracted are discursive distancing, strategic borrowing and fortifying boundaries. Inductive themes include hostile sexism, suicidality and clown world
11. Konutgan (58)	*N* = 139 28 self-identified incels *M* Age = 24.78 41.01% male	N/A	*N* = 111 Non-incels	Positively significant correlation between entitlement and frustrated mating needs (FMN) (*r* = .26) Entitlement and acceptance of modern myths about sexual aggression (AMMSA) (*r* =. 61) Entitlement and hostility towards women (*r* = .48) FMN and hostility towards women (*r* =. 37) FMN and AMMSA (*r* = .40) AMMSA and hostility towards women (*r* =.69)
12. Sparks et al. (20)	*N* = 107 67 self-identified incels	N/A	*N* = 103 non-incel male students	Incels reported higher scores of depression (*M* = 25.23, *SD* = 4.93) and anxiety (*M* = 18.43, *SD* = 4.52) than non-incels (*M* = 15.92, *SD* = 6.60, *M* = 15.83, *SD* = 4.27 respectively). Significant differences reported between emotional support than non-incels (*F* = 23,201, *p* < 0.001, *d* = 0.88) and loneliness (*F* = 62.630, *p* < 0.001, *d* = 1.47). Significant differences between incels and non-incels for sexual entitlement (*F* = 7.453, *p* < 0.001, *d* = 0.50) and belief in female deceptiveness (*F* = 30.636, *p* < 0.001, *d* = 1.01)

### Quality assessments

I. CASP

The CASP tool evaluated the qualitative data (*N* = 2). Although none of the studies were without risk of bias, especially with the inclusion of self-report methodology, all studies were considered to be of good quality, where each study was rated “low”, “high”, or “unclear of bias”. The item regarding the consideration of ethical issues which was unclear for most studies. Despite this, no studies were excluded for their quality (see **Table 5**).

**Table 5:** CASP tool for qualitative sources.

Appraisal criteria items	9	10
Clear aims?	Yes	Yes
Appropriate methodology?	Yes	Yes
Appropriate research design?	Yes	Yes
Appropriate recruitment strategy?	Yes	Yes
Appropriate data collection?	Yes	Yes
Considered participants-researcher relationship?	Yes	N/A
Considered ethical issues?	Yes	N/A
Rigorous data analysis?	Yes	Yes

II. JBI Checklist for Analytical Cross-Sectional Studies

The JBI analytical cross-sectional tool was used for the cross-sectional studies (*N* = 7) (1, 2, 3, 4, 5 11, 12), all deemed of good quality according to the checklist (see **Table 6**). This was despite the inclusion of self-report methodology and no mention of confounding variables. The checklist (check **Appendix 3**) posed eight questions, with responses categorised as yes, no, unclear or not applicable.

**Table 6:** JBI tool for cross-sectional studies.

Appraisal criteria items	1	2	3	4	5	11	12
Clear inclusion criteria?	No	No	No	No	No	No	No
Clear study subjects and setting?	Yes	Yes	Yes	Yes	Yes	Yes	Yes
Valid and reliable measurement of exposure/s?	Yes	Yes	Yes	Yes	Yes	Yes	Yes
Confounding factors identified?	No	No	No	No	No	No	No
Strategies for confounding factors?	N/A	N/A	N/A	N/A	N/A	N/A	N/A
Valid and reliable measurement of outcome/s?	Yes	Yes	Yes	Yes	Yes	Yes	Yes
Appropriate statistical analysis	Yes	Yes	Yes	Yes	Yes	Yes	Yes

While all studies clearly outlined their sampling methods and setting, selection criteria for participants were less clear. Reliable, objective and valid measures were used for all studies, with Cronbach’s alpha indicating good to excellent reliability (Check **Appendix 4**).

Most studies included students, recruited by offering course credits or as partially fulfilling course requirements (2, 3, 4, 5, 11, 12), while one study offered monetary incentives (2). Some studies advertised on incel-dominated social media platforms (1, 3, 11, 12).

Appropriate statistical analyses were used, including but not limited to Pearson’s correlation, multiple regression analyses and path analyses to test the relationships between numerous variables (1, 3, 4, 11).

III. JBI critical appraisal tool for case reports

Two studies were assessed using the JBI tool for case reports (see **Table 7**). Centonze et al. (77) clearly outlined the patient’s demographic characteristics, diagnosis, symptomatic presentation and the intervention principle used. The authors stated the timeline of the treatment, stating symptomatic improvement after two years. Weinberg's (27) case report case report was also assessed as good quality according to the checklist as they clearly outlined the patient’s demographic characteristic, diagnosis and symptoms. However, the timeline of the patient’s history and the post intervention clinical condition was not clearly described, therefore the findings should be interpreted cautiously in light of this. Overall, the paper was found to be of good quality and was not excluded.

**Table 7:** JBI tool for case reports.

Appraisal criteria items	6	8
Demographic characteristics?	Yes	Yes
Patient’s history clearly presented as timeline?	Yes	Unclear
Current clinical condition described?	Yes	Yes
Diagnostic assessments?	No	No
Interventions?	Yes	Yes
Post-intervention clinical condition described?	Yes	Unclear
Adverse events described?	Yes	No
Takeaway lessons?	Yes	Yes

IV. JBI Critical Appraisal Tool for Expert Opinion

Dimaggio’s (25) paper was found to be of excellent quality as they clearly outlined their opinion, which was congruent with the opinions present in the literature of PN and NPD. The interests of the relevant population, pathological narcissists, were the central focus of the article.

### Outcomes

#### Maladaptive thinking and cognition

Bonfa-Araujo and Filho (74) found a positive correlation between dichotomous thinking and the dark triad, highlighting the cognitive patterns associated with PN. They found that ERS significantly positively correlated with dichotomous thinking, but not with narcissism or psychopathy. However, a significant positive correlation was observed between ERS and Machiavellianism. Further analysis using multiple linear regressions showed that dichotomous thinking had a weaker association with narcissism compared to its stronger link with Machiavellianism.

Campbell et al. (75) conducted two experiments. In the first (N = 160), individuals with higher narcissism scores demonstrated a self-serving bias, taking credit for success to the discredit of their partner. Conversely, non-narcissists exhibited an other-serving bias, taking less credit for success and more responsibility for failure. In the second experiment (*N* = 64), where tasks were completed independently, both narcissists and non-narcissists took more responsibility for success than failure, indicating self-enhancement. Participants who succeeded viewed their outcomes as more important than those who failed.

Similarly, Sparks et al. (20) explored responses to romantic rejection and dating attitudes and found that non-incels are more likely to externalise blame (*M* = 9.74, *SD* = 4.35) than incels (*M* = 7.93, *SD* = 5.02), with a small significant difference between the two groups (*F* = 4.475, *p* < 0.05, *d* = 0.39).

Mason and Deshong (33) found that specific traits of the different domains of the 3FM were associated with different types of RNTs. They used a path analysis to simultaneously estimate numerous regression models. All of the measures used and their internal consistencies can be found in the appendices (see **Appendix 4**). The researchers reported narcissistic neuroticism a significant predictor as it positively associated with general rumination, worry and catastrophising. Self-centred antagonism was also reported to be a significant predictor as it positively associated with anger rumination and catastrophising, but negatively associated with worry. The NPI score only had a significant but negative correlation with worry. Overall, these results highlight the differential impacts of narcissistic domains on various forms of RNTs. Sparks et al. (20) also found that incels also engaged in rumination, specifically self-critical rumination (*M* = 32.85, *SD* = 6.45) more than non-incels (*M* = 26.62, *SD* = 7.45), with a significant difference between the two groups (*F* = 22.035, *p* < 0.001, *d* = 0.87), highlighting the use of maladaptive thinking in incels.

Ziegler-Hill et al. (76) found that specific narcissistic traits were individually associated with different maladaptive schemas (see **Appendix 5** for scales). All traits of narcissism were associated with the entitlement schema. VN was positively associated with subjugation. Significant associations between GN and several early maladaptive schemas were identified. Specifically, GN was positively correlated with mistrust (β = .31, *p* <.001), entitlement (β = .12, *p* <.05), self-sacrifice (β = .18, *p* <.01), and unrelenting standards (β = .13, *p* <.05), while it was negatively associated with insufficient self-control (β = −.14, *p* <.05). These schemas were significant predictors of GN. Additionally, VN was significantly predicted by positive associations with mistrust (β = .20, *p* <.001), subjugation (β = .19, *p* <.01), dependence (β = −.16, *p* <.05), abandonment (β = .23, *p* <.001), and entitlement (β = .13, *p* <.05).

Ziegler-Hill and colleagues also found gender differences as men scored higher than women on the following measures of narcissism: leadership/authority (*t*[440] = 3.25, *p* <.001), superiority/arrogance (*t*[440] = 3.94, *p* <.001), exploitation/entitlement (*t*[440] = 2.34, *p* <.05), and grandiose narcissism (*t*[440] = 2.17, *p* <.05). Gender differences for early maladaptive schemas were also reported as men reported higher scores than women for emotional deprivation (*t*[440] = 2.63, *p* <.01), emotional inhibition (*t*[440] = 3.25, *p* <.001), mistrust (*t*[440] = 2.71, *p* <.01), defectiveness (*t*[440] = 4.05, *p* <.001), subjugation (*t*[440] = 3.23, *p* <.001), and failure to achieve (*t*[440] = 2.74, *p* <.01).

Dimaggio (25) identified that maladaptive relational schemas significantly affect individuals with PN, who often possess paradoxical views of themselves and others, concealing feelings of inferiority, coupled with expectations of admiration and a presentation of superiority. This instability in self-concept and fluctuating self-esteem hinders their ability in forming and maintaining social relationships. Dimaggio (25) argues this is exacerbated by a limited capacity for self-reflection and a tendency to intellectualize, which are consistent themes in the literature (27). Empathic impairments and difficulties with theory of mind further complicate interpersonal relationships. Weinberg (27) notes that individuals with PN are less inclined to engage in self-understanding and reflection, as their primary focus is on affirming their self-perceptions. These dysfunctional cognitive patterns significantly contribute to the challenges faced by individuals with PN.

### Entitlement

Sparks et al. (20) assessed sexual entitlement using a subscale of the sexual narcissism scale and found that incels (*M* = 11.26, *SD* = 5.11) had a higher score than non-incels (*M* = 9.09, *SD* = 3.82), with a large effect size (*d* = 0.50).

Konutgan (58) examined the relationship between psychological factors and misogynistic beliefs (*N* = 139). Multiple regression analyses exhibited significant positive correlations between sexual entitlement and frustrated mating needs (FMN). Sexual entitlement also had a positive correlation with misogynistic beliefs, including acceptance of rape myths and hostility towards women (check **Appendix 4** for scales). Sexual entitlement and FMN were significant predictors of both rape myth acceptance and hostile attitudes towards women. Specifically when regressing the Acceptance of Modern Myths About Sexual Aggression Scale on entitlement and frustrated mating needs, these factors together explained 44% of the variance in rape myth acceptance (*R²* = .44, *F*(147) = 5 6.04, *p* <.001) and 29% of the variance in hostility towards women (*R²* = .29, *F*(148) = 30.37, *p* <.001), highlighting the significant contribution of sexual entitlement and FMN on these misogynistic beliefs.

### Social support and social rejection

Sparks et al. (20) observed that incels reported significantly lower levels of perceived social support (*M* = 25.22, *SD* = 11.69) compared to non-incels (*M* = 40.35, *SD* = 10.68), with a large, statistically significant difference (*F* = 54.285, p < 0.001, *d* = 1.37). Furthermore, incels exhibited higher levels of loneliness (*M* = 24.33, *SD* = 3.33) than non-incels (*M* = 18.59, *SD* = 4.18), again with a significant difference between the groups (*F* = 62.630, *p* < 0.001, *d* = 1.47). The study also revealed that only one-third of incels reported having a friend outside of their online interactions, further outlining the lack of social support amongst incels (20).

Ksinan and Vazsonyi's (32) study (*N* = 536) utilised a survey involving five scales, measuring narcissism and its subscales, GN and VN, preference for online social interaction (POSI), social anxiety and social self-efficacy (see **Appendix 4** for a list of the scales). Statistical analyses including Spearman’s Rho, path analyses and multiple linear regressions revealed a significant positive association between VN and social anxiety (*r* = .53, *p* < 0.01), compared to a significant negative association between GN and social anxiety (*r* = -.25, *p* < 0.01). POSI positively correlated with social anxiety (*r* = .49, *p* < 0.01) and negatively with social self-efficacy (*r* = -.39, *p* < 0.001). VN also positively correlated with POSI (*r* = .39, *p* < 0.01), whereas GN negatively correlated with this preference (*r* = -.09, *p* < 0.05). Bootstrapping analyses also revealed that VN had significant indirect effects through POSI on both social anxiety and self-efficacy, whereas GN did not. Thus, individuals with higher levels of VN are more likely to experience social anxiety and prefer online social interactions over face-to-face interactions.

Daly and Reed (10) identified significant themes of social rejection, wherein participants attributing their ostracism to their perceived deficiencies in personality or physical appearance. Consequently, incels seek social support within online incel forums. One participant, Stephen, remarked “Society thinks short guys are just weak losers … they always get mocked or rejected for that” (10, p. 22). Another interviewee, Mark, described the cyclical nature of this dynamic: incels, experiencing social rejection, increasingly turn to online communities for support, which in turn reinforces their identification with a stigmatised group, therefore culminating in social isolation. The incel participants further expressed that loneliness is a significant contributing factor to the development of depressive symptoms and suicidal ideation, with another interviewee stating “I was extremely suicidal just for the fact that I had no girlfriend” and characterised his experience as “pure suffering” (10, p. 27).

### Distorted gender attitudes: hegemonic masculinity, hybrid masculinity and misogyny

Sparks et al. (20) found that incels endorsed more misogynistic attitudes than non-incels, especially in response to romantic rejection, as they scored higher on scales of belief in female deceptiveness and sexual entitlement. Similarly, Konutgan (58) found positive correlations between FMN and AMMSA, and with hostility towards women. They also found correlations between AMMSA and hostility towards women.

Daly and Reed (10) identified a central theme of masculinity challenges among incels, where victimhood is constructed as a “badge of honour” and becomes a dominant expression of masculinity. This allows incels to reclaim a sense of masculine identity, which they feel is compromised by their perceived inadequacies in masculinity, appearance, mental health, and personality. The authors also observed “shitposting”, often using discriminatory and provocative language, as a means of locally reproducing their perception of masculinity, while simultaneously feeling marginalised for lacking masculinity on a broader scale. One interviewee described shitposting as a way to “channel their anger and sadness” (10, p. 26), suggesting it may also serve as an emotional outlet. This participant also noted that incels post misogynistic content to seek attention and validation from fellow forum users, to reclaim their masculinity through homosocial currencies.

Another key theme is incels viewing themselves as subhuman, positioned at the bottom of a social hierarchy compared to women and Chads, who possess the most power through their ability to engage in sexual activity (10). This belief system underpins the misogynistic attitudes prevalent within the incel community. Using the hegemonic masculinity framework to explain incel related gender attitudes, Daly and Reed (10) outline that incels adopt ‘masculine’ practices, like shitposting, while discursively distancing themselves from other dominant masculine practices by embracing their incel identity. These findings align with the hybrid masculinity framework discussed by Glace et al. (15).

Glace et al. (15) argue that incels perform hybrid masculinity through three key themes: discursive distancing without actually relinquishing control, borrowing elements from other marginalised cultures, and the fortifying of boundaries within hegemonic masculinity by maintaining its power structure. They found that incels exhibit a conflicted relationship with masculinity, promoting hegemonic masculine values through endorsing misogynistic beliefs, while attempting to distance themselves from these same values by portraying themselves as victims. Despite complaints of their failures in obtaining relationships, incels attempt to reclaim their masculinity by claiming they would hypothetically reject women’s sexual advances due to their perceived promiscuity, revealing contradictions within incel ideology and hybrid masculinity. The researchers also observed that incels often belittle other ‘non-masculine’ men, for example, referring to less masculine men as “soyboys” due to the link between soy and oestrogen.

Glace et al. (15) also identified several inductive themes, including hostility toward women, manifested in practices such as slut-shaming, degradation, and dehumanisation by reducing women to their sexuality. Incels justify these misogynistic attitudes by claiming its adherence to traditional gender roles and citing false rape claims. Incels argue that accusations of misogyny are directed primarily at unattractive men, claiming that women are comfortable engaging in relations with “Chads”, but falsely accuse incels of rape, labelling them as “creepy” (15, p. 294).

### Coping strategies

Maladaptive coping strategies, including perfectionism, rumination, and avoidance, are often employed to manage psychological distress (77). Dimaggio (25) emphasises the mitigation of such maladaptive strategies as a key therapeutic goal for treating PN. Sparks et al. (20) state that incels are more likely to endorse maladaptive coping strategies, such as self-distraction, social withdrawal and self-blame, compared to non-incels, and exhibit lower levels of adaptive coping strategies like positive reframing (see **Table 8**). Furthermore, Sparks et al. (20) suggest that incels’ lack of social support and heightened loneliness may exacerbate their engagement in maladaptive coping behaviours, as they have fewer outlets for managing romantic and sexual frustration.

**Table 8:** Sparks et al.’s (20) findings.

Coping strategy	Incels M(SD)	Non-incels (M, SD)	F	Cohen’s d
Self-blame	6.54 (1.68)	5.23 (1.92)	15.335, p < 0.001	.71
Behavioural disengagement	5.08 (1.99)	3.11 (1.57)	39.009, p < 0.001	1.31
Venting	4.92 (1.77)	3.63 (1.47)	19.657, p < 0.001	.81
Self-distraction	6.50 (1.76)	5.81 (1.76)	4.558, p < 0.05	.39
Positive reframing	4.31 (2.16)	5.38 (1.72)	9.626, p < 0.01	.56
Emotional support	2.90 (1.26)	4.35 (1.85)	23.201, p < 0.001	.88

Cohen’s d values:.1 = small effect size,.3 = moderate effect size,.5 = large effect size.

Incels describe their participation in online forums as “… a means of coping …”, providing social support when without real-life support (10, p. 26). However, incels acknowledge that this engagement may worsen their social rejection by reinforcing their identification with a marginalised group, thus contributing to further ostracization (10). This aligns with Ksinan and Vazsonyi (32) findings where individuals with higher levels of VN are more likely to experience social anxiety and prefer online social interactions.

### Therapeutic recommendations

To develop effective treatments for PN, an integrative therapeutic approach is recommended, drawing on principles from metacognitive interpersonal therapy (MIT), transference-based psychotherapy (TFT), psychoanalytically oriented psychotherapy (POP), and mentalisation-based therapy (MBT) (7, 8). These approaches specifically target the mechanisms of change required for PN, addressing issues related to self-reflection, empathy, theory of mind, maladaptive schemas, and coping strategies (6).

Centonze et al. (77) and Dimaggio (25) identify several factors contributing to interpersonal difficulties in PN, including a lack of empathy, poor theory of mind, reliance on maladaptive coping strategies, problematic self and interpersonal schemas, a tendency to intellectualise, limited self-awareness, and diminished personal agency.

The therapeutic alliance is a key determinant of treatment outcomes for PN. Weinberg (27) reported that the quality of the clinician-client relationship is the strongest predictor of therapeutic success, as it is associated with lower client drop-out rates and reduced clinician burnout (6, 7, 8). Given that PN often encompasses power struggles (6), alliance ruptures must be addressed to strengthen the therapeutic relationship. To improve this alliance, Centonze et al. (77) and Dimaggio (25) suggest interventions to enhance theory of mind and empathy, which are critical. A positive therapeutic relationship can subsequently serve as a model for other interpersonal relationships. However, the provocative characteristics of individuals with PN may evoke negative emotions in clinicians, like irritation. Clinicians may feel as though they must “walk on eggshells,” thereby increasing the risk of therapeutic stalemates (27, p.2). To mitigate these challenges, Weinberg (27) advises clinicians to adopt strategies managing these difficult behaviours and avoid co-created impasses. Recommendations include setting realistic treatment goals, addressing collusion in prolonged states of mutual idealisation or devaluation, preventing the patient from controlling the therapy process, and ensuring the appropriate termination of therapy when necessary.

Experiential techniques, for example, chair play, are also recommended to enhance self-reflection and theory of mind, thus developing more functional interpersonal schemas. Experiential work allows a safe environment for individuals with PN to explore healthier responses to events, without fear of judgement, to reduce experiences of shame and humiliation (6). Weinberg (27) emphasises enhancing reflective functioning as a critical mechanism of change in psychotherapy for PN, particularly given the this population’s tendency to intellectualise self-reflection (7). Additionally, facilitating mourning—grieving unmet wishes, needs, and fantasies—is emphasised as a crucial therapeutic goal, conducted through addressing maladaptive coping strategies (8).

Centonze et al.’s (77) case study involving client, Laura, who presented comorbid generalised anxiety disorder, depression, complicated grief and covert narcissism. After two years of metacognitive interpersonal therapy, which emphasised experiential work and the repair of alliance ruptures, Laura showed significant improvement. Her symptomatic presentation was reduced, as well as the use of problematic coping strategies, such as perfectionism, reduced social withdrawal, healthier self-image and increased self-reflection.

### Narrative summary

Research has shed light on the lived experiences of incels, highlighting critical themes such as societal rejection, loneliness, and social isolation (9, 10, 12). A correlation exists between inceldom and adverse mental health outcomes, with the lack of social support a potential moderating variable (12). This absence of support and the use of maladaptive coping mechanisms, such as social withdrawal, may deprive incels of healthy outlets to express frustrations related to peer, societal, and romantic rejections (3, 9, 12). These frustrations often stem from feelings of entitlement and may fuel misogynistic attitudes (11). Misogynistic beliefs include the acceptance of rape myths, hostility toward women, and the use of derogatory and regressive language (9, 10, 11). However, incels stress that not all incels subscribe to such extreme beliefs. Shame also often manifests as depression, suicidal ideation, and a strict adherence to the nihilistic BlackPill ideology (9).

The themes identified in incels are also evident in pathological narcissists, who often develop maladaptive schemas early in life, characterised by unrelenting standards, entitlement, and a lack of empathy (5). To manage the subsequent psychological distress, they employ maladaptive coping strategies such as social isolation, rumination, and perfectionism (2, 3, 4, 6, 7). Distorted thinking is common, as narcissists engage in self-enhancement strategies to preserve their self-image of superiority, often at others’ expense (2). Incels also display distorted thinking in social comparisons, attributing their romantic failures to perceived deficiencies in their appearance, personality and status, often comparing themselves unfavourably to “Chads” (9, 10).

Therapy for individuals with PN aims to enhance self-reflection and interpersonal functioning. Given the interpersonal difficulties that narcissists face, therapy can be challenging for both client and therapist. Experienced practitioners recommend integrating techniques from various psychological therapies, such as MIT and MBT, to make treatments accessible to all clinicians. Such techniques, including experiential techniques, aim to dismantle maladaptive schemas, fostering healthier schemas and coping mechanisms to mitigate negative emotional states such as frustration and annihilation. Therapy also facilitates self-reflection, helping individuals recognise and articulate their internal experiences. Clinicians receive guidance on managing therapeutic challenges, such as avoiding stigmatisation and creating a therapeutic environment conducive to client growth (5, 6, 7, 8). This guidance also helps clinicians manage the challenges of working with this difficult population and reduce the risk of burnout (6, 8).

## Discussion

The systematic review aimed to synthesise the current literature on PN and inceldom to draw comparisons between the two populations, raising a critical question for future research: Could treatment strategies developed for PN be applicable for incels, thus potentially mitigating incel-related harmful behaviours? Twelve studies were selected for their adherence to the selection criteria, utilising diverse methodologies, including interviews, surveys, and case studies. The literature consistently identifies misogyny, toxic masculinity, and aggrieved entitlement as central themes within inceldom, alongside loneliness, social rejection and maladaptive coping. Similarly, salient themes in PN include maladaptive cognition and dysfunctional coping mechanisms. Given the lack of empirically supported interventions, this study also examined therapeutic strategies to address these challenges.

Participants consistently exhibited dysfunctional cognition, including maladaptive schemas and distorted thinking patterns, potentially leading to erroneous judgments (78). One study examined the relationship between various conceptualisations of narcissism and repetitive negative thinking styles (RNT) (33). Although focusing only on four types of RNT, it found that worry, general rumination, anger rumination, and catastrophising were positively correlated with narcissistic neuroticism. Yet only anger rumination and catastrophising were positively associated with self-centred antagonism, highlighting the complex interplay between maladaptive cognition and different facets of narcissism.

Dichotomous thinking - another dysfunctional cognitive pattern - positively, albeit non-significantly, correlates with overt narcissism (74, 78) and linked to perfectionism, a common maladaptive cognitive strategy among narcissists (44, 45, 75, 77). While dichotomous thinking may temporarily reduce anxiety by simplifying several stimuli to two outcomes only, it ultimately results in erroneous processing biases (44). Further research could offer particular insights into why grandiosely narcissistic individuals often pursue perfectionism and perceive anything less than perfect as failure.

Dichotomous thinking is also evident among incels. Hegemonic and hybrid masculinity frameworks help elucidate the gender-related attitudes exhibited by incels. The incel view of masculinity is marked by paradoxical, polarised thinking. Hegemonic masculinity typically encompasses traits such as physical strength, the role of the breadwinner, heterosexual sexual success, and a balance of stoicism and aggression in emotional regulation (79). Incels, however, perceive themselves as subhuman due to self-perceived inferiorities in such qualities compared to Chads, who embody hegemonic masculinity (57). This comparative thinking style mirrors that of narcissistic individuals, who perform downward social comparisons to preserve self-esteem (38). Incels, in contrast, make upward comparisons with those they perceive as superior, leading to feelings of narcissistic malignant envy, shame and humiliation (28, 38). These emotions, coupled with maladaptive coping behaviours such as anger rumination, can precipitate extreme reactions, including violence, as a means to reclaim perceived lost masculinity.

On a more localized level, however, incels construct an “inverted hierarchy” (57, p. 244). This allows them to reclaim masculinity through valorising their victimhood as incels, while belittling men they consider non-masculine, hence reflecting the hybrid masculinity framework (15). This is akin to the downward comparisons made by pathological narcissists. Nevertheless, underlying feelings of shame and humiliation often manifest as a sense of defeat (15). One incel participant expressed, “…we’re angry from feelings of rejection, depression, and frustration … it makes suicide a daily and constant staple of my thoughts” (10, p. 27). The correlation between narcissistic rivalry and depression is well-documented, with social support identified as a mediating factor—a critical consideration given incels’ pervasive lack of social support (80, 81). Though this weaponisation of victimhood does not represent all incels, one remarked, “They think I want to be an Incel and that’s so wrong because no man wants to be an Incel” (10, p.23). Ultimately, the paradox in incels’ view of gender - distance themselves from masculinity while still valuing masculine power - reflects an attempt to satisfy their feelings of entitlement to sex while maintaining their victimhood.

Incels display paradoxical and polarised gender attitudes toward both men and women. They objectify and desire women while simultaneously expressing misogynistic aggression. Incels attribute power to women by positioning them as gatekeepers of sex and relationships, yet associate femininity with weakness and derogatorily label sexually active women as promiscuous (10, 15). Their misogynistic beliefs are linked to measures of sexual entitlement and sexual narcissism (20, 58, 82), and are deeply rooted in incels’ self-perceptions of inadequacy (15). Incels justify their acceptance of rape myths by claiming sexual assault only occurs when the perpetrator is not a Chad, arguing that women are sexually promiscuous with them. Misogynistic practices, like slut-shaming through shitposting, contradict incels’ belief in a biologically determined social hierarchy, which should theoretically mitigate the blame on women (15, 58). However, incels’ objectify women, reducing them to their bodies, to which they feel entitled access to, irrespective of biological determinism (10). Additionally, incels condemn women’s use of makeup/cosmetic surgery, despite engaging in similar self-enhancement practices like ‘looksmaxxing’, to improve relationship prospects. This prompts inquiries and necessitates clarification of boundaries of the incel identity, as it remains unclear what practices are considered acceptable or are condemned (15, 16).

Despite their nihilistic self-identification as “BlackPilled”, incels exhibit a pronounced sense of entitlement to sex and relationships, viewing female sexuality as women’s primary value (15, 58). This sense of entitlement is especially evident among young, white males (21). When denied what they perceive as their inherent right, the resulting incongruence between their ideal and actual selves triggers shame and humiliation (58, 79). This aggrieved entitlement parallels Beck’s self-concept theory, which posits that this incongruence can incite negative affect such as depression and low self-esteem (24, 83). Such emotional distress may fuel misogynistic beliefs (15, 58), potentially culminating in incel-related violence (22, 57, 84). An illustrative example is Elliot Rodger, whose masculinity crisis, exacerbated by bullying and perceived inadequacy, ultimately led to violence as a last resort to strengthen his fragile masculinity (22, 79). Both GN and VN, along with entitlement rage, mediate the relationship between fragile masculinity and aggression (79), underscoring the importance of addressing these traits when discussing the prevention of incel-related harmful behaviours.

Entitlement is also a central feature of PN, significantly predicted by various maladaptive schemas, such as social isolation, failure and abandonment (76). In PN, entitlement often manifests as an expectation of external admiration to affirm one’s uniqueness (31). Ziegler-Hill et al. (76) identified varying relationships between facets of narcissism and early maladaptive schemas. All measures of narcissism correlated positively with the entitlement schema, with GN strongly correlating with schemas like unrelenting standards and mistrust, while VN correlated with mistrust, subjugation, dependence, and abandonment (76). These findings align with previous research, demonstrating the differing relationships between different narcissistic traits and early maladaptive schemas (78, 85), but overall, still a key concept of PN.

Cognitive impairments in self-reflection and theory of mind are consistently observed among individuals with PN (25, 35, 77). Pathological narcissists often intellectualise their reflections, resulting in what can be described as pseudo-reflection. Nuances in empathic functioning exist across different presentations of PN. Those with VN typically exhibit a greater capacity for metacognitive reflection, enabling them to contemplate their thoughts and feelings; but struggle with metacognitive insight, which involves a clear understanding of these feelings (41). In contrast, individuals with GN display deficiencies in both reflection and insight, highlighting distinctions in narcissistic presentations. This empathy deficit may be erroneously perceived by narcissists as strategically advantageous, enhancing productivity through quicker decision-making and increased risk-taking (86). However, this impaired theory of mind often leads to social consequences, including the exploitation and neglect of others’ emotions. Though individuals with PN may find empathy overwhelming as they not only grapple with processing their own emotions but also attempt to comprehend others’ feelings (86).

Interpersonal difficulties is a recurring theme in both incel culture and PN. Many incels report lacking friendships outside their online incel communities (20). Social support is crucial for buffering the impact of adverse events, such as romantic rejection (20), so its absence may leave incels without healthy outlets for their frustrations. Adopting The General Strain Theory (87) could shed light on why this population may resort to aggression. It posits that “strains,” or stressors, trigger negative emotions. For incels, chronic isolation, whether self-imposed or due to ostracization, can exacerbate negative emotions like resentment. Combined with chronic sexlessness, this isolation may constitute the strain that drives incels toward violence (22, 57, 82, 84). The accumulation of negative emotions fosters a sense of inevitability, leading to a perceived need for “corrective action” through violence. Rodger exemplified this, framing his actions as driven by “anger, sadness, and hatred” in a “war against cruel injustice” (5, p. 1). This is particularly alarming in post-COVID, as online platforms have become more accessible sources of social support (10, 20). Vulnerable narcissists, in particular, often prefer online interactions due to heightened sensitivity to rejection and social anxiety (32, 88). While the internet can provide a semblance of social support, it can also reinforce negative beliefs. One incel remarked, “I always felt depressed, but after the BlackPill, I feel happy, and I feel free now because I don’t need to worry about getting a girlfriend anymore and no more getting rejected by women for my looks” (10, p. 24). This underscores the need for healthier coping mechanisms among younger generations. Encouraging group activities that offer safe peer interactions could disrupt the harmful cycle of social isolation, preference for online interactions, and mental health issues.

Individuals with PN and incels share barriers to cognitive and behavioural change needed to enhance well-being and life satisfaction, particularly a marked reluctance to seek and maintain mental health support. One incel remarked “no matter how many times incels go to see [a] therapist, they’d still be sexually unattractive, and that’s the core problem from my views,” further asserting that therapy would “not end their real problems” (10, p. 25). Similarly, individuals with PN often struggle with shame, perfectionism, and persistent boredom, potentially preventing engagement in therapy (27). It is therefore crucial to equip clinicians with appropriate therapeutic tools to maintain engagement, thereby reducing dropout rates.

The primary objectives of therapy for these populations include symptomatic reduction and improving relational patterns. Various therapeutic approaches have been recommended for PN, including MIT, DBT, TFT and CBT (25, 27, 77), with techniques across these approaches contributing to therapeutic change. However, the suggested therapeutic principles are often based on case reports, particularly with comorbid anxiety and mood disorders (20, 77). Therefore, this must be considered when generalising treatment strategies for individuals with PN, especially for those without comorbidities. For example, Dimaggio (25) emphasises the importance of addressing a lack of agency, particularly in VN, though this is often associated with comorbid depression. Given the interconnected nature of the pathological components within this population, strategies such as behavioural tasks, typically recommended for improving coping or relational patterns, may also serve to reinforce agency.

Given the impaired cognitive reflection in PN, processes that enhance theory of mind, such as metacognitive reflection and insight, must be incorporated into treatment. MBT principles are particularly effective in this context, as they promote mentalising feelings such as shame, humiliation, and inadequacy (86). Clients are encouraged to recognise when their mentalizing capacity is impaired, such as during “concrete modes”—a state in which they rigidly perceive their thoughts and feelings as absolute truths, disregarding alternative perspectives. This mirrors the incel community’s adherence to the BlackPill ideology, which asserts that they are romantically doomed and beyond help (89).

Another critical component of treatment for these populations is facilitating mourning, where clients confront the reality of unmet fantasies or expectations. Enhancing reflective functioning can support this process (27). This is vital, as setbacks and challenges can negatively impact various aspects of their lives, including relationships, work, motivation, decision-making, and overall well-being (41). Therefore, facilitating mourning can buffer the negative impacts of adversities experienced by these populations.

Furthermore, skills training, a core principle of DBT, can equip clients with tools for distress tolerance, interpersonal effectiveness, and mentalisation for everyday real-life situations (27, 86). Experiential techniques, consistently recommended by clinicians, enhance recognition of internal states, thereby fostering reflective abilities and reducing intellectualisation (25, 27, 77). Techniques such as guided imagery or chair work offer a safe environment for clients to practice healthy behavioural responses to challenging events (77).

Another critical issue in treating PN is the managing alliance ruptures, to prevent treatment impasses (27). By negotiating the therapeutic contract firmly, as practiced in TFP, a collaborative approach can be maintained and reduces power struggles. This ensures that the client does not dominate the therapeutic process and effectively manages countertransference (25, 77, 86). A case study in this review demonstrated significant progress after two years of utilising the aforementioned strategies (77).

### Limitations and directions for future research

The primary limitation of this review is the significant gap in research concerning the psychological variables underlying inceldom. Although this study only draws theoretical parallels between the attitudes, beliefs, and behaviours of inceldom and PN, the findings primarily serve as a foundation for future empirical research on psychological factors of inceldom.

The review also considers the inherent challenges of studying the incel community. Notably, few incel participants were featured (e.g., 10, 58), as incels often avoid outsiders and remain as an anonymous population, limiting the generalisability of findings (58). Researchers must be cautious, as incels may feel judged, particularly when asked about misogynistic attitudes, which could deter future participation. Konutgan (58) recommends direct communication with incels, thereby potentially easing tension and fostering greater participation, as Daly and Reed (10) did, though they had to consider that their interviewer - a young, Asian woman - may have affected the findings. Furthermore, only one study employed a standardised instrument to measure inceldom, the Incel Traits Scale (20, 59). The Narcissistic Personality Inventory was also utilised (60, 62, 90, 91), though it primarily assesses non-pathological narcissism and may not capture the nuances of PN (63, 92, 93). Therefore, using standardised, empirical measures would be advantageous in reducing potential biases and facilitating greater comparability of findings.

### Conclusion

To conclude, the intersection of PN and incel ideology highlights similarities in both phenomena being deeply rooted in maladaptive cognitive patterns, distorted self-perceptions and impaired interpersonal skills. PN, characterised by overarching traits of superiority and entitlement, is conceptualised by different models which exhibit nuances in its different presentations. Likewise, the incel ideology displays patterns of aggrieved entitlement and unstable emotional regulation. Both share presentations of similar traits such as superiority, entitlement and low self-esteem, as well as the adoption of maladaptive coping strategies like social withdrawal. Given the overlap between these constructs, it would be incredibly valuable to investigate the potential application of intervention principles for PN to address incel attitudes, and to mitigate the rise of incel-related incidents, particularly in light of the distressing rising rates of femicide globally.

## Data Availability

The original contributions presented in the study are included in the article/**Supplementary Material**. Further inquiries can be directed to the corresponding author/s.
