# Supplementary figures and images for: Pathological narcissism and inceldom: can the application of treatment principles for PN help reduce the rise of incel-related incidents?
Source: Front Psychiatry. 2025 May 30;16:1513719. doi: 10.3389/fpsyt.2025.1513719 (PMC12163613; doi:10.3389/fpsyt.2025.1513719)

**Appendix 1**

*CASP checklist for Qualitative Sources*


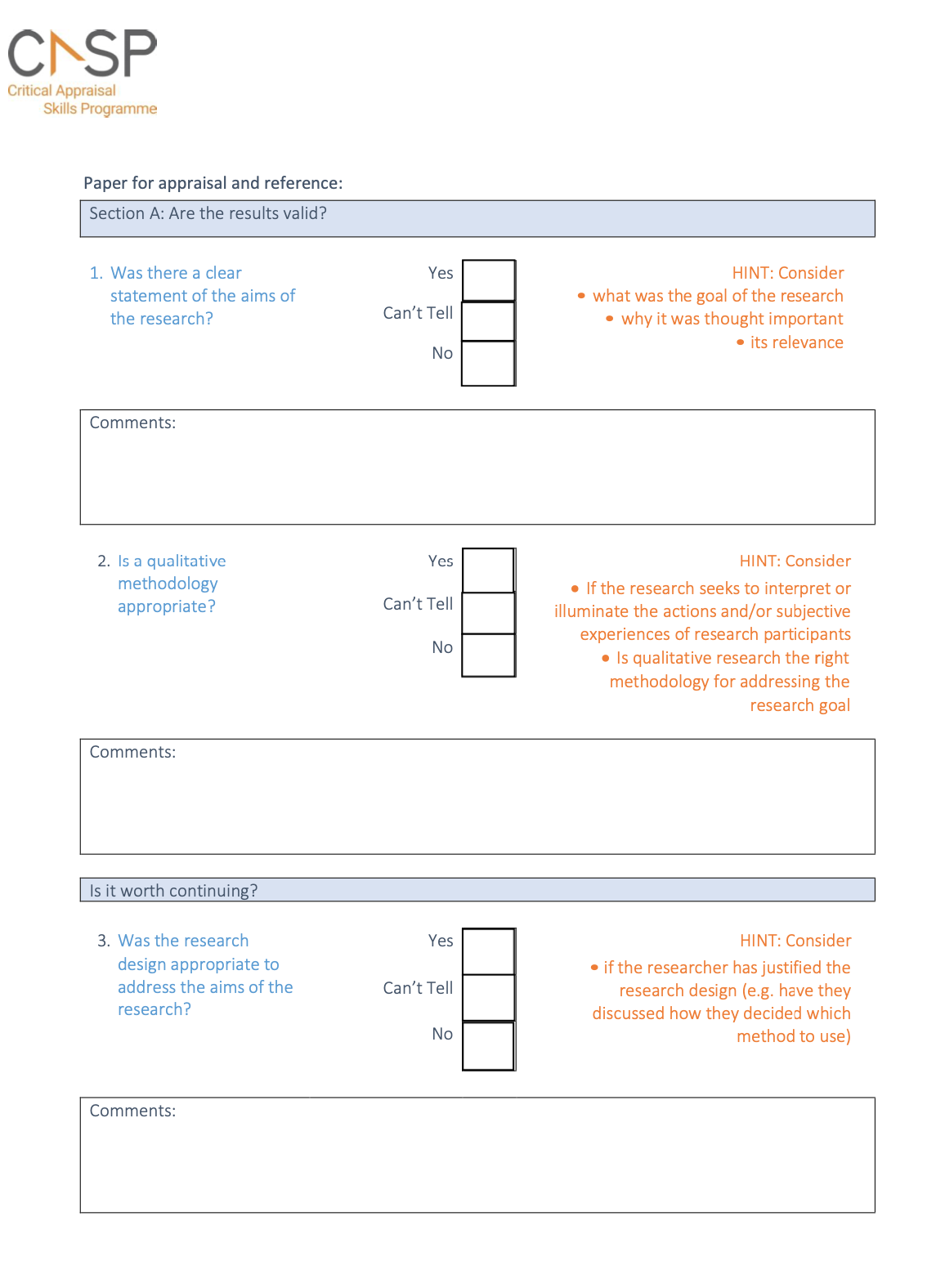

Supplement: Supplementary file 1 [file Table1.docx]

**Appendix 2**

*JBI Checklist for Cross-Sectional Study*


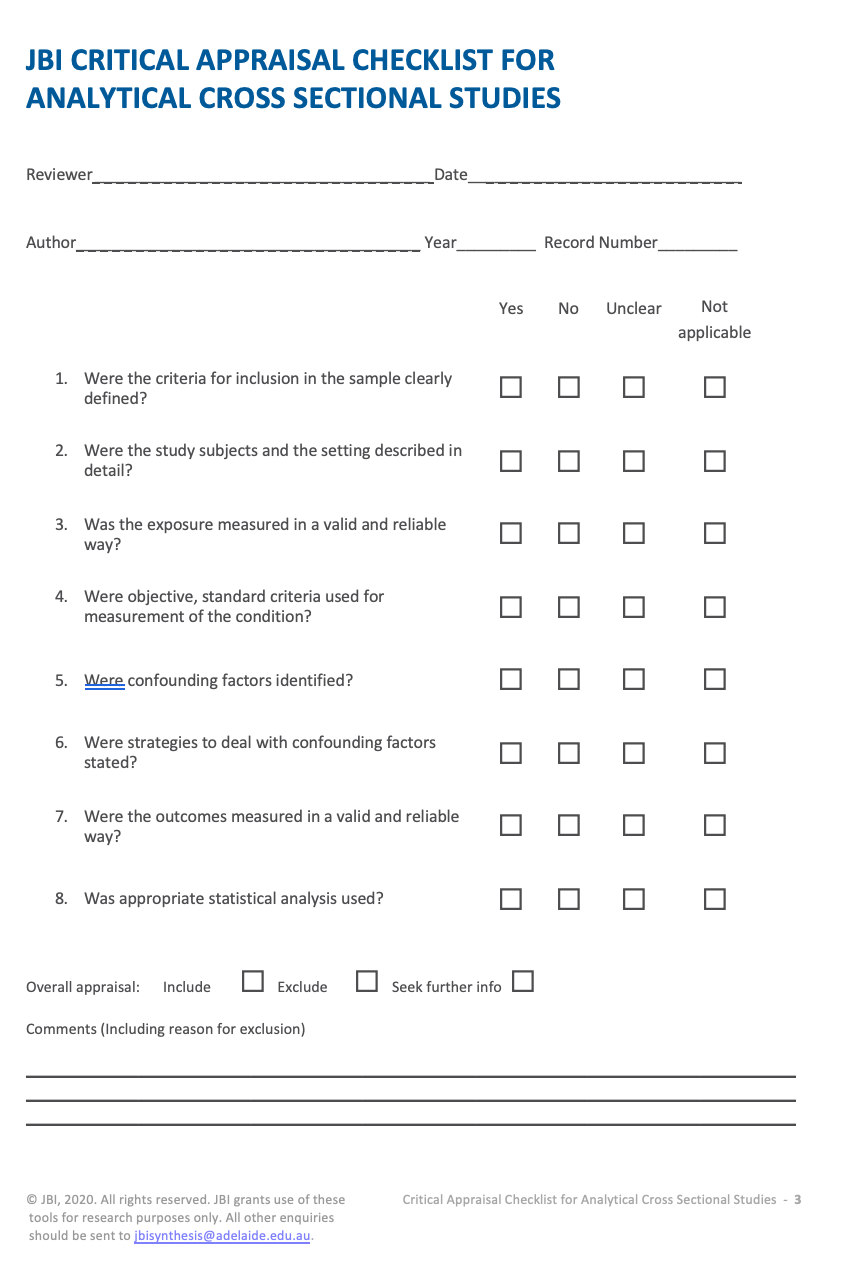

Supplement: Supplementary file 2 [file Table2.docx]

**Appendix 3**

*JBI Checklist for Case Reports*


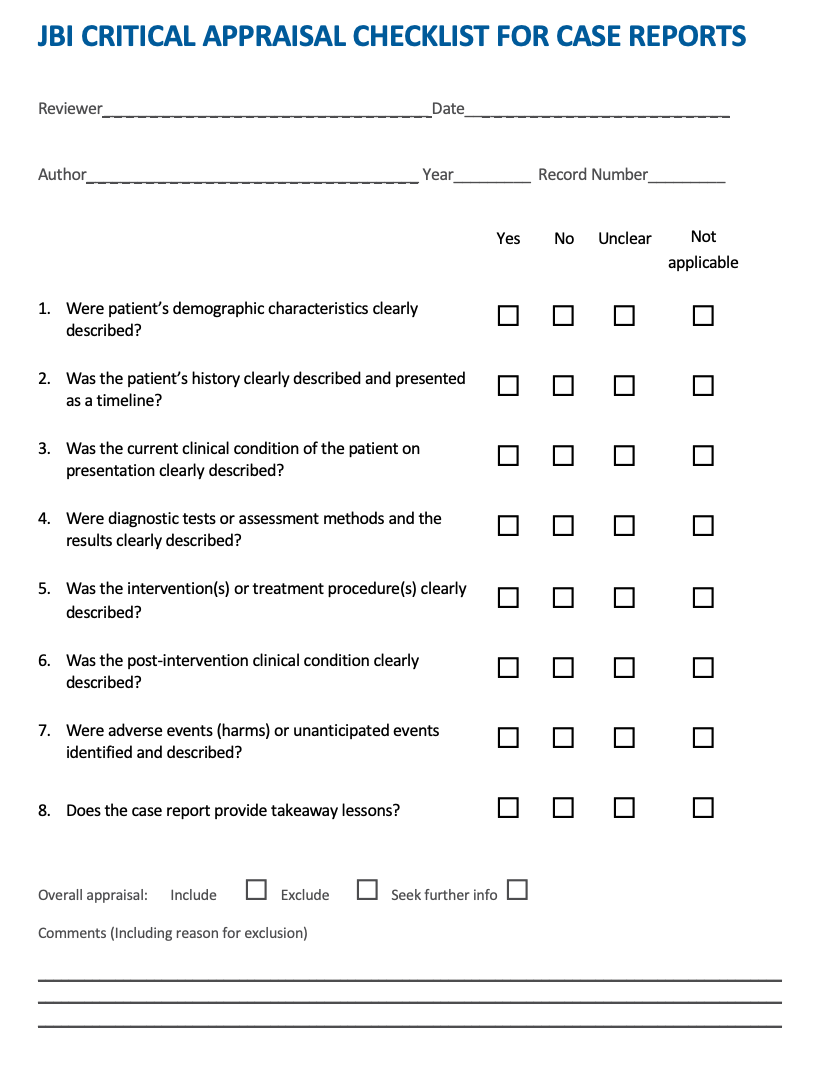

Supplement: Supplementary file 3 [file Table3.docx]

**Appendix 4**

*JBI Checklist for Textual Evidence: Expert Opinion*

*
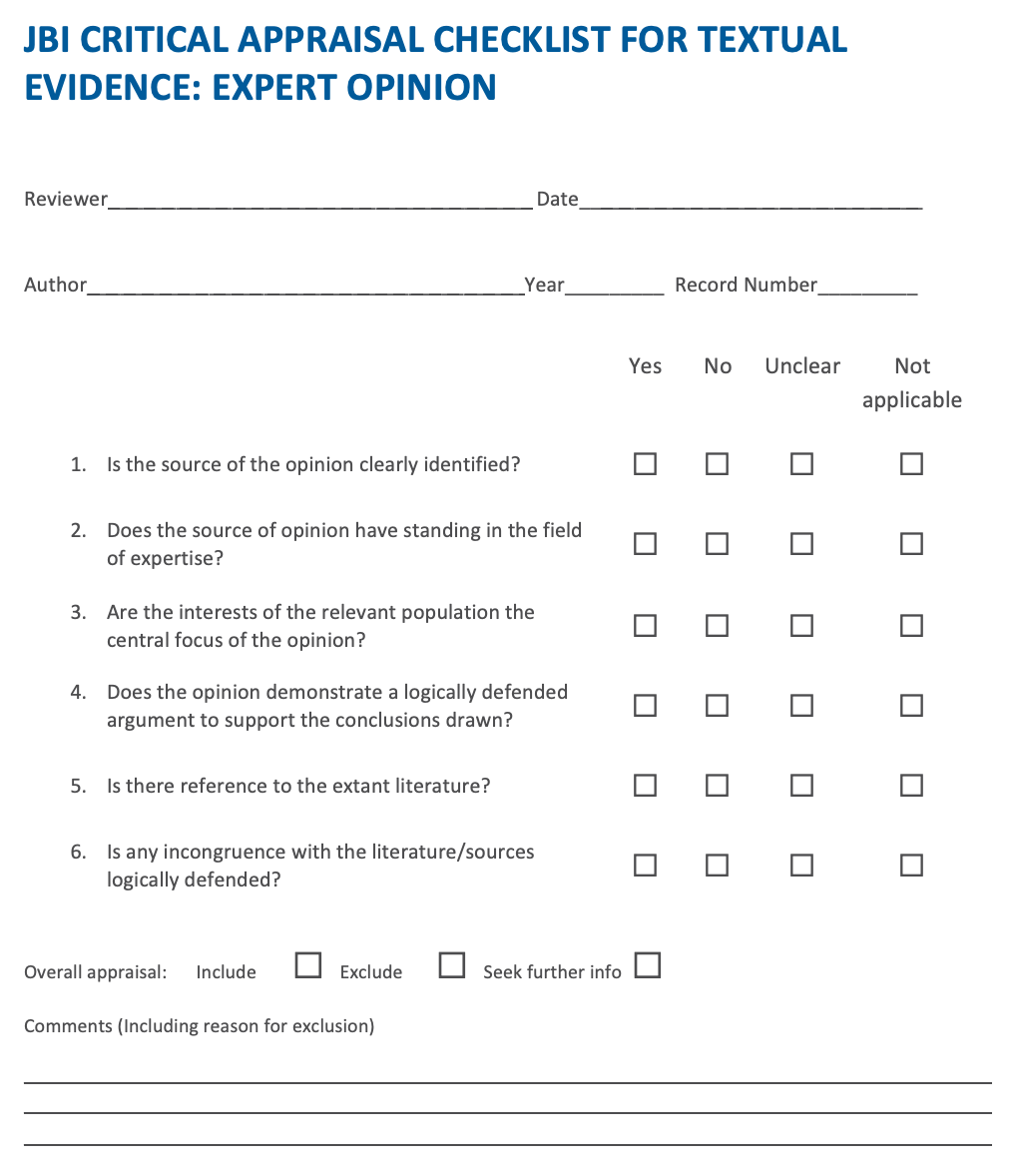
*

Supplement: Supplementary file 4 [file Table4.docx]
