# Supplementary material for: Pathological narcissism and inceldom: can the application of treatment principles for PN help reduce the rise of incel-related incidents?
Source: Front Psychiatry. 2025 May 30;16:1513719. doi: 10.3389/fpsyt.2025.1513719 (PMC12163613; doi:10.3389/fpsyt.2025.1513719)
Supplement: Supplementary file 5 [file Table5.docx]

**Appendix 5**

*Internal consistency for measures*

| **Study** | **Outcome measures** | | **Internal consistency measure** |
| --- | --- | --- | --- |
|  | **Outcome** | **Instrument** |  |
| **Bonfá-Araujo & Hauck Filho, 2021** | Extreme Response style (ERS)  Dichotomous thinking  Dark Triad Traits | Response pattern Scale (RPS; Greenleaf, 1992).  Dichotomous Thinking Inventory (DTI; Oshio,  2012)  Short dark triad (SD3; Jones & Paulhus, 2014). | Ωₜ = .81  Ωₜ = .92  Machiavellianism: Ωₜ = .80  Psychopathy: Ωₜ = .77  Narcissism: Ωₜ = .73 |
| **Campbell et al 2000** | Narcissism  Self-esteem | Narcissistic Personality Inventory (NPI; Raskin & Hall, 1979)  Rosenberg Self-Esteem Inventory (RSE; Rosenberg, 1965) | Adequate reliability and validity  Adequate reliability and validity |
| **Konutgan, 2020** | I.Sexual entitlement *  II.Misogynistic beliefs  III.Frustrated Mating needs (FMN) | I.Hanson Sex Attitudes scale (Hanson, Gizarelli & Scott, 1994)  Sexual Narcissism Scale (Widman & Mcnulty, 2010)  II.Hostility Towards Women (Hanson et al., 1994)  Acceptance of Modern Myths About Sexual Aggression (Gerger, Kley, Bohner & Siebler, 2007)  III.Hanson Sex Attitudes questionnaire (Widman & Mcnulty, 2010) | Sexual entitlement: α = .88  α = 0.72  α = 0.9  α = 0.96 |
| **Ksinan & Vazsonyi, 2016** | Grandiose Narcissism  Vulnerable Narcissism  Preference for Online Social Interactions (POSI)  Social Anxiety  Social Self-Efficacy | NPI-16 scale ([Ames, Rose, & Anderson, 2006](https://www.sciencedirect.com/science/article/pii/S0191886916300174#bb0005)).  Hypersensitive Narcissism scale ([Hendin & Cheek, 1997](https://www.sciencedirect.com/science/article/pii/S0191886916300174#bb0105))  Preference for online social interaction subscale of Generalized Problematic Internet Use 2 scale ([Caplan, 2010](https://www.sciencedirect.com/science/article/pii/S0191886916300174#bb0065))  Social Interaction Anxiety Scale (SIAS; [Mattick & Clarke, 1998](https://www.sciencedirect.com/science/article/pii/S0191886916300174#bb0145))  Scale of Perceived Social Self-Efficacy (SSE; [Smith & Betz, 2000](https://www.sciencedirect.com/science/article/pii/S0191886916300174#bb0220)) |  |
| **Mason & DeShong (2023)** | Narcissism  Repetitive Negative Thinking Styles | Five Factor Narcissism inventory – Short Form (FFNI-SF; Sherman et al., [2015](https://link.springer.com/article/10.1007/s10862-023-10085-1#ref-CR58))  NPI-21 (Svindseth et al., [2008](https://link.springer.com/article/10.1007/s10862-023-10085-1#ref-CR62))  Anger Rumination Scale (ARS; Sukhodolsky et al., [2001](https://link.springer.com/article/10.1007/s10862-023-10085-1#ref-CR60))  Cognitive Emotional Regulation Questionnaire (CCERQ; Garnefski et al., [2001](https://link.springer.com/article/10.1007/s10862-023-10085-1#ref-CR17))  Penn State Worry Questionnaire (PSWQ; Meyer et al., [1990](https://link.springer.com/article/10.1007/s10862-023-10085-1#ref-CR38)) | Agentic extraversion: α = 0.77  Narcissistic Neuroticism: α = 0.74  Self-centred antagonism: α = 0.77)  α = 0.80  Anger rumination: α = .93  General rumination = α = .75  Catastrophising = α = .76  α = .95 |

*Note*. * Both Sexual entitlement and Misogynistic beliefs were positively loaded above 0.5 when factor analyses for these scales were conducted to ensure they were valid measures of the variables

(Table continued)

| **Study** | **Outcome Measures** | | | **Internal consistency of measures/subscales** |
| --- | --- | --- | --- | --- |
|  | **Outcome** | | **Instrument** |  |
| **Sparks et al (2023)** | Attachment styles | | State adult attachment scale (Gillath et al., [2009](https://link.springer.com/article/10.1007/s12144-023-04275-z#ref-CR25)) | Secure (α = 0.91)  Anxious (α = 0.87)  Avoidant (α = 0.82) |
|  | Anxious and depressive symptoms | | Hospital anxiety and depression scale (Zigmond & Snaith, [1983](https://link.springer.com/article/10.1007/s12144-023-04275-z#ref-CR80)) | Depression (α = 0.77)  Anxiety (α = 0.80). |
|  | Fear of singlehood | | Fear of being single (Spielmann et al, [2013](https://link.springer.com/article/10.1007/s12144-023-04275-z#ref-CR68)) | (α = 0.89) |
|  | Self-esteem | | Single-item self-esteem scale (Robins et al, [2001](https://link.springer.com/article/10.1007/s12144-023-04275-z#ref-CR60)) | Not provided |
|  | Externalisation of blame | | Externalisation of blame scale (Kelly & Aunspach, [2020](https://link.springer.com/article/10.1007/s12144-023-04275-z#ref-CR41)) | (α = 0.82) |
|  | Support for group dominance and hiererchies | | Short social dominance orientation scale (Pratto et al., [2013](https://link.springer.com/article/10.1007/s12144-023-04275-z#ref-CR58)) | (α = 0.69) |
|  | Quality of themselves as mate | | Mate value scale (Edlund and Sagarin, [2014](https://link.springer.com/article/10.1007/s12144-023-04275-z#ref-CR22)) | (α = 0.92) |
|  | Loneliness | | Social and emotional loneliness scale (De Jong Gierveld & Van Tillburg, [2006](https://link.springer.com/article/10.1007/s12144-023-04275-z#ref-CR17)) | (α = 0.71) |
|  | Sexual narcissism | | Sexual entitlement subscale of sexual narcissism scale (Widman and McNulty, 2009) | (α = 0.81) |
|  | Self-critical rumination | | Self-critical rumination scale (Smart et al., [2016](https://link.springer.com/article/10.1007/s12144-023-04275-z#ref-CR65)) | (α = 0.92) |
|  | Subscription to incel ideology | | Incel traits scale (Scaptura & Boyle, 2019) | (α = 0.95) |
|  | Coping strategies | | Brief cope scale (Carver, [1997](https://link.springer.com/article/10.1007/s12144-023-04275-z#ref-CR13)) | Not provided |
|  | Perceived social support | | Multidimensional scale of perceived social support (Zimet et al., [1988](https://link.springer.com/article/10.1007/s12144-023-04275-z#ref-CR81)) | (α = 0.92) |
|  | Belief that women are sexually manipulative | | Belief in female sexual deceptiveness (Rogers et al., [2015](https://link.springer.com/article/10.1007/s12144-023-04275-z#ref-CR62)) | (α = 0.95) |
| **Ziegler-Hill et al (2011)** | Narcissism  Early Maladaptive Schemas | NPI (NPI; [Raskin and Hall, 1979](https://www.sciencedirect.com/science/article/pii/S0005791610000704#bib36))  Pathological Narcissism Inventory (PNI, [Pincus et al., 2009](https://www.sciencedirect.com/science/article/pii/S0005791610000704#bib34))  Young Schema Questionnaire – Short Form (YSQ-SF; [Young, 1998](https://www.sciencedirect.com/science/article/pii/S0005791610000704#bib52)) | | leadership (α = .75),  self-absorption (α = .71)  superiority (α = .70)  entitlement (α = .68)  grandiosity (α = .86)  vulnerability (α = .95)  Emotional deprivation (α = .91)  Emotional inhibition (α = .89),  Mistrust (α = .92)  Social isolation (α = .93)  Defectiveness (α = .94)  Subjugation (α = .89)  Dependence (α = .83)  Failure to achieve (α = .95)  Vulnerability to harm (α = .90)  Abandonment (α = .93)  Enmeshment (α = .83)  Insufficient self-control (α = .85)  Entitlement (α = .81),  Self-sacrifice (α = .86)  Unrelenting standards (α = .88) |
