# Supplementary material for: Pathological narcissism and inceldom: can the application of treatment principles for PN help reduce the rise of incel-related incidents?
Source: Front Psychiatry. 2025 May 30;16:1513719. doi: 10.3389/fpsyt.2025.1513719 (PMC12163613; doi:10.3389/fpsyt.2025.1513719)
Supplement: Supplementary file 6 [file Table6.docx]

**References**

Caplan, S. E. (2010). Theory and measurement of generalized problematic Internet use: A two-step approach. *Computers in Human Behavior*, *26*(5), 1089–1097. https://doi.org/10.1016/j.chb.2010.03.012

Carver, C. S. (1997). You want to measure coping but your protocol’s too long: Consider the Brief COPE. *International Journal of Behavioral Medicine*, *4*(1), 92–100. https://doi.org/10.1207/s15327558ijbm0401_6

Edlund, J. E., & Sagarin, B. J. (2014). The Mate Value Scale. *Personality and Individual Differences*, *64*, 72–77. https://doi.org/10.1016/j.paid.2014.02.005

Garnefski, N., Kraaij, V., & Spinhoven, P. (2001). Negative life events, cognitive emotion regulation and emotional problems. *Personality and Individual Differences*, *30*(8), 1311–1327. https://doi.org/10.1016/s0191-8869(00)00113-6

Gerger, H., Kley, H., Bohner, G., & Siebler, F. (2007). The acceptance of modern myths about sexual aggression scale: development and validation in German and English. *Aggressive Behavior*, *33*(5), 422–440. https://doi.org/10.1002/ab.20195

Gierveld, J. D. J., & Tilburg, T. V. (2006). A 6-Item Scale for Overall, Emotional, and Social Loneliness. *Research on Aging*, *28*(5), 582–598. https://doi.org/10.1177/0164027506289723

Gillath, O., Hart, J., Noftle, E. E., & Stockdale, G. D. (2009). Development and validation of a state adult attachment measure (SAAM). *Journal of Research in Personality*, *43*(3), 362–373. https://doi.org/10.1016/j.jrp.2008.12.009

Greenleaf, E. A. (1992). Measuring Extreme Response Style. *Public Opinion Quarterly*, *56*(3), 328. https://doi.org/10.1086/269326

Jones, D. N., & Paulhus, D. L. (2014). Introducing the Short Dark Triad (SD3): A Brief Measure of Dark Personality Traits. *Assessment*, *21*(1), 28–41. https://doi.org/10.1177/1073191113514105

KARL HANSON, R., GIZZARELLI, R., & SCOTT, H. (1994). The Attitudes of Incest Offenders. *Criminal Justice and Behavior*, *21*(2), 187–202. https://doi.org/10.1177/0093854894021002001

Kelly, C. R., & Aunspach, C. (2020). Incels, Compulsory Sexuality, and Fascist Masculinity. *Feminist Formations*, *32*(3), 145–172. https://doi.org/10.1353/ff.2020.0044

Mattick, R. P., & Clarke, J. Christopher. (1998). Development and validation of measures of social phobia scrutiny fear and social interaction anxiety. *Behaviour Research and Therapy*, *36*(4), 455–470. https://doi.org/10.1016/s0005-7967(97)10031-6

Meyer, T. J., Miller, M. L., Metzger, R. L., & Borkovec, T. D. (1990). Development and validation of the penn state worry questionnaire. *Behaviour Research and Therapy*, *28*(6), 487–495. https://doi.org/10.1016/0005-7967(90)90135-6

Pratto, F., Çidam, A., Stewart, A. L., Zeineddine, F. B., Aranda, M., Aiello, A., Chryssochoou, X., Cichocka, A., Cohrs, J. C., Durrheim, K., Eicher, V., Foels, R., Górska, P., Lee, I-Ching., Licata, L., Liu, J. H., Li, L., Meyer, I., Morselli, D., & Muldoon, O. (2013). Social Dominance in Context and in Individuals. *Social Psychological and Personality Science*, *4*(5), 587–599. https://doi.org/10.1177/1948550612473663

Robins, R. W., Hendin, H. M., & Trzesniewski, K. H. (2001). Measuring Global Self-Esteem: Construct Validation of a Single-Item Measure and the Rosenberg Self-Esteem Scale. *Personality and Social Psychology Bulletin*, *27*(2), 151–161. https://doi.org/10.1177/0146167201272002

Rosenberg, M. (1965). Society and the adolescent self-image. *Social Forces*, *44*(2), 255. https://doi.org/10.2307/2575639

Smart, L. M., Peters, J. R., & Baer, R. A. (2015). Development and Validation of a Measure of Self-Critical Rumination. *Assessment*, *23*(3), 321–332. https://doi.org/10.1177/1073191115573300

Smith, H. M., & Betz, N. E. (2000). Development and Validation of a Scale of Perceived Social Self-Efficacy. *Journal of Career Assessment*, *8*(3), 283–301. https://doi.org/10.1177/106907270000800306

Spielmann, S. S., MacDonald, G., Maxwell, J. A., Joel, S., Peragine, D., Muise, A., & Impett, E. A. (2013). Settling for less out of fear of being single. *Journal of Personality and Social Psychology*, *105*(6), 1049–1073. https://doi.org/10.1037/a0034628

Sukhodolsky, D. G., Golub, A., & Cromwell, E. N. (2001). Development and validation of the anger rumination scale. *Personality and Individual Differences*, *31*(5), 689–700. https://doi.org/10.1016/s0191-8869(00)00171-9

Svindseth, M. F., Nøttestad, J. A., Wallin, J., Roaldset, J. O., & Dahl, A. A. (2008). Narcissism in patients admitted to psychiatric acute wards: its relation to violence, suicidality and other psychopathology. *BMC Psychiatry*, *8*(1). https://doi.org/10.1186/1471-244x-8-13

Widman, L., & McNulty, J. K. (2009). Sexual Narcissism and the Perpetration of Sexual Aggression. *Archives of Sexual Behavior*, *39*(4), 926–939. https://doi.org/10.1007/s10508-008-9461-7

Young, J. E. (1998). Young Schema Questionnaire--Short Form. *Cognitive Therapy Centre*. https://doi.org/10.1037/t12644-000

Zigmond, A. S., & Snaith, R. P. (1983). The Hospital Anxiety and Depression Scale. *Acta Psychiatrica Scandinavica*, *67*(6), 361–370. https://doi.org/10.1111/j.1600-0447.1983.tb09716.x

Zimet, G. D., Dahlem, N. W., Zimet, S. G., & Farley, G. K. (1988). The Multidimensional Scale of Perceived Social Support. *Journal of Personality Assessment*, *52*(1), 30–41. https://doi.org/10.1207/s15327752jpa5201_2
